# Paraneoplastic pemphigus involved oral mucosa only: a case report and literature review

**DOI:** 10.3389/fmed.2025.1498800

**Published:** 2025-01-27

**Authors:** Xixi Yu, Mei Yang, Caixia Wang, Wanchun Wang, Tingting Zhu, Yang Liu

**Affiliations:** ^1^Department of Oral Mucosa, Qingdao Stomatological Hospital Affiliated to Qingdao University, Qingdao, China; ^2^Department of Oral Medicine, Peking University School and Hospital of Stomatology, Beijing, China

**Keywords:** paraneoplastic pemphigus, neoplasms, autoimmune disease, autoantibodies, oral mucosa

## Abstract

Paraneoplastic pemphigus (PNP) is a rare and potentially fatal autoimmune disease that primarily affects the mucocutaneous tissues. Here we present the clinical features of a patient with the lichenoid variant of PNP involving the oral mucosa only. The patient exhibited clinical manifestations resembling both lichen planus and pemphigus, yet these lesions remained unresponsive to corticosteroid therapy. A chest computed tomography (CT) scan revealed Castleman disease. Following surgical removal of the tumor, the patient’s oral mucosa healed completely, with no recurrence observed in a 4-year follow-up period. These findings emphasize the importance of considering PNP as a potential diagnosis in patients with refractory oral erosions displaying lichen planus-like and pemphigus-like features, particularly when unresponsive to corticosteroid therapy. A thoracic-abdominal-pelvic CT scan is recommended to facilitate accurate diagnosis and appropriate treatment.

## Introduction

Paraneoplastic pemphigus (PNP), also known as paraneoplastic autoimmune multiorgan syndrome (PAMS), is a rare autoimmune disease characterized by complex mucocutaneous lesions and associated with potential neoplasms (1). Non-Hodgkin lymphoma (NHL) has been identified as the most common associated neoplasm (38.6%) globally, followed by chronic lymphocytic leukemia (18.4%) (2). However, in China, Castleman disease (CD) is the predominant associated neoplasm, accounting for 56% of cases (3). The pathogenesis of PNP is still not fully understood, but autoantibodies targeting various tissues have been implicated.(4). Clinically, PNP manifests in various forms, including lichen planus-like, pemphigus-like, erythema multiforme-like, bullous pemphigoid-like, toxic epidermal necrolysis-like, and chronic graft-versus-host disease-like presentations (2). Mucositis may be the only manifestation of PNP in some cases (5).

## Case presentation

**FIGURE 1:**
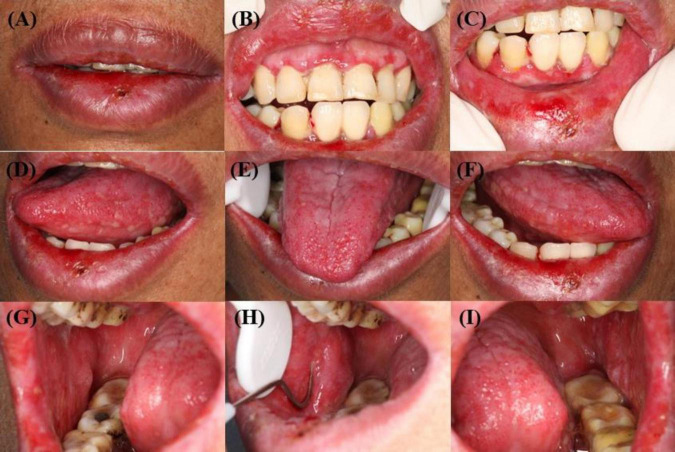
**(A–I)** Mucosal physical examination: diffuse erosive stomatitis. **(A–C)** Scattered vesicles on the red mucous membranes of the upper and lower lips. **(D–F)** Extensive vesicles and congestion of the mucous membranes of the ventral and dorsal part of the tongue bilaterally. **(G–I)** Extensive vesicles and exudate on the buccal mucous membranes.

**FIGURE 2:**
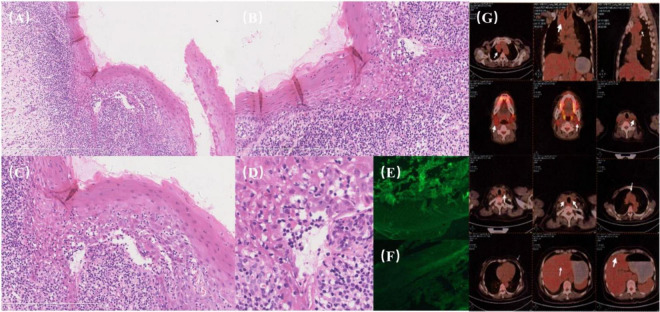
**(A–G)** Histopathological and imaging findings. **(A)** Dense lymphocytic infiltration within the epithelium (“mossy appearance”) with liquefactive degeneration of basal cells, indicative of interfacial dermatitis. **(B)** Basal cell liquefaction accompanied by dense lymphocytic infiltration. **(C)** Clarification needed: “Untwisting of the vertebral column”—consider specifying histopathological or structural relevance, as this phrase seems out of context for histology. **(D)** Cellular degeneration and keratinization surrounded by peripheral lymphocytic infiltration. **(E,F)** Direct immunofluorescence (DIF): Negative for intercellular deposits of IgG and C3. **(G)** PET/CT imaging: a soft tissue mass on the right side of the upper mediastinum and trachea with mild FDG metabolic activity.

A 57-year-old woman presented with a 3-month history of progressive oral mucosal erosion. She denied experiencing fever, rash, joint pain, chest tightness or skin lesions but reported a 5 kg weight loss. Oral examination revealed extensive white reticulation with reticulations, superficial erosions and erythema across oral mucosa (Figures 1A–I). A biopsy of the right buccal mucosa showed dense infiltration of epithelial lymphocytes within the lamina propria and liquefied degeneration of basal cells consistent with interfacial dermatitis (Figures 2A–D). Direct immunofluorescence (DIF) revealed no deposition of immunoglobulin G (IgG) or Complement 3 (C3) in the epidermal intercellular spaces or basement membrane zone (Figures 2E, F). Indirect immunofluorescence (IIF) using rat bladder epithelium also showed no deposition of IgG. Enzyme-linked immunosorbent assays (ELISAs) for anti-desmoglein (Dsg 1 and 3) and BP antigen 180 (BP180 and BP230) were negative. Based on these findings, the patient was initially diagnosed with erosive oral lichen planus, with a possible diagnosis of paraneoplastic pemphigus (PNP).

The patient was treated with prednisolone at 20 mg/day for 7 days, but her symptoms worsened. Computed tomography (CT) and PET/CT scans revealed a soft tissue mass on the right side of the upper mediastinum and trachea with mildly increased FDG metabolism, suggesting the possibility of reactive lymph node inflammation (Figure 2G).

The patient’s pre-treatment frozen serum was successfully located and retested for antibodies against plakins (envoplakin and periplakin), yielding positive results. Surgical resection of the mass was performed, and histopathological examination confirmed the diagnosis of Castleman’s disease.

**FIGURE 3:**
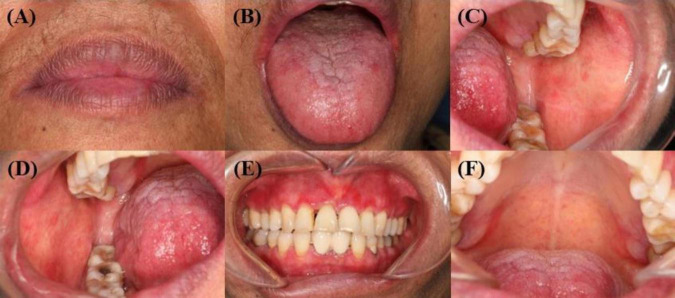
**(A–F)** Complete healing of oral mucosa after surgery. **(A)** The original vesicles of the red lip mucosa healed completely from before. **(B)** The vesicles of the dorsal tongue mucosa and the ventral tongue mucosa healed completely from before. **(C–D)** The original vesicles of the buccal mucosa healed completely from before, and the exudation disappeared. **(E–F)** Other mucosal erosions of the oral cavity have completely healed from the previous condition.

Postoperatively, the patient was treated with oral prednisolone at 20 mg/day. Her intraoral symptoms resolved completely within 1 month after surgery, after which the prednisolone dosage was gradually tapered to a maintenance dose of 2 mg/day, which she continued for 1.5 years (Figure 3). At the 4-year follow-up, the patient remained asymptomatic with no recurrence of oral mucosal lesions.

## Discussion

Paraneoplastic pemphigus (PNP), also known as paraneoplastic autoimmune multiorgan syndrome (PAMS), is a rare and life-threatening autoimmune bullous disease affecting the skin and mucous membranes (6). PNP has a high mortality rate (up to 90%) and a 5-year overall survival rate of approximately 38% (7). It is frequently associated with underlying tumors, particularly lymphoproliferative disorders (8). CD is the most common neoplasm associated with PNP in China (3) and South Korea (9), while non-Hodgkin lymphoma is the predominant neoplasm globally. This suggests a potential relationship between the type of underlying neoplasm and ethnicity. The pathogenesis of PNP involves both cell-mediated and humoral autoimmunity, although the precise mechanism remains unclear. Autoantibodies targeting plakin family proteins and desmosomal cadherins play a central role in the disease process (7, 10, 11).

The symptoms of PNP manifest in diverse forms (4, 12). Refractory oral mucositis is the hallmark of PNP and (13)often persists throughout the disease course (1). Other mucous sites including the pharynx, larynx, esophagus, eye (14, 15), genital and intestinal (5) may also be affected. Skin lesions typically follow mucosal involvement and are often extensive, affecting the trunk, head, and neck (16–18). In China, 90% of PNP patients present with skin lesions, most commonly lichen planus-like (37%) or pemphigus-like (20%) lesions (3). In our case, the patient’s disease was limited to oral mucosal involvement without skin lesions, which may reflect the benefits of early diagnosis and surgical excision of the underlying neoplasm.

There are currently no widely accepted diagnostic criteria for PNP (1). Anhalt et al. first proposed diagnostic criteria in 1990, but these have since been refined as understanding of the disease evolves (6). According to the 2023 guidelines from the European Academy of Dermatology and Venereology (EADV), the presence of autoantibodies to envoplakin, desmoplakin, periplakin, or A2ML1 is highly specific for PNP. In this case, the patient initially presented with oral mucosal lesions resembling both lichen planus and pemphigus, accompanied by histological findings of intraepidermal acantholysis, keratinocyte necrosis, and basal lamina degeneration. The detection of serum antibodies against periplakin and envoplakin confirmed the diagnosis of PNP. While DIF, IIF, and ELISA tests for Dsg1, Dsg3, BP180, and BP230 were negative, the sensitivity and specificity of these tests for PNP can vary. For example, the sensitivity of DIF for intercellular IgG and C3 deposits ranges from 41 to 83% (19), while autoantibodies to Dsg3 and Dsg1 are identified in 65 and 25% of PNP cases, respectively (20, 21). Approximately 40% of PNP sera are positive for BP180 (5, 22). The sensitivity and specificity of antibodies against periplakin and envoplakin are 74.2 and 96.3%, respectively, and 83 and 91%, respectively (23, 24). Moreover, DSC-3, DSC-2, and DSC-1 antibodies have been reported in 60.8, 41.2, and 18.6% of PNP cases, respectively (25). These findings highlight the variability in diagnostic markers and the importance of combining clinical, histological, and serological data to confirm PNP. Surgical excision of the primary tumor is a pivotal aspect of PNP management. In Chinese patients, concomitant Castleman disease or thymoma is frequently observed, and tumor removal often results in rapid improvement of skin and mucosal lesions. However, in some cases, symptoms persist despite tumor resection. The prognosis for Chinese PNP patients is generally more favorable compared to other populations.

High-dose corticosteroids remain the first-line treatment for PNP (26, 27), but mucosal lesions often respond poorly to corticosteroids and other immunosuppressive agents, such as cyclosporine, cyclophosphamide, azathioprine, and mycophenolate mofetil (27–30). In our case, the patient’s oral mucosal lesions did not respond to corticosteroid therapy, prompting further investigation. Following surgical removal of the tumor, the intraoral lesions resolved completely, and no recurrence was observed during a 4-year follow-up period.

This case underscores the importance of early diagnosis, multidisciplinary management, and timely surgical intervention in PNP. By promptly identifying and excising the underlying neoplasm, it is possible to achieve favorable outcomes, even in cases with refractory mucosal lesions.

## Data Availability

The original contributions presented in this study are included in this article/supplementary material, further inquiries can be directed to the corresponding authors.
